# Metagenomic and whole-genome analysis reveals new lineages of gokushoviruses and biogeographic separation in the sea

**DOI:** 10.3389/fmicb.2013.00404

**Published:** 2013-12-24

**Authors:** Jessica M. Labonté, Curtis A. Suttle

**Affiliations:** ^1^Department of Microbiology and Immunology, University of British ColumbiaVancouver, BC, Canada; ^2^Department of Earth, Ocean and Atmospheric Sciences, University of British ColumbiaVancouver, BC, Canada; ^3^Department of Botany, University of British ColumbiaVancouver, BC, Canada; ^4^Canadian Institute for Advanced Research, University of British ColumbiaVancouver, BC, Canada

**Keywords:** biogeography, ssDNA viruses, *Microviridae*, *Gokushovirinae*, virus diversity, ocean viruses

## Abstract

Much remains to be learned about single-stranded (ss) DNA viruses in natural systems, and the evolutionary relationships among them. One of the eight recognized families of ssDNA viruses is the *Microviridae*, a group of viruses infecting bacteria. In this study we used metagenomic analysis, genome assembly, and amplicon sequencing of purified ssDNA to show that bacteriophages belonging to the subfamily *Gokushovirinae* within the *Microviridae* are genetically diverse and widespread members of marine microbial communities. Metagenomic analysis of coastal samples from the Gulf of Mexico (GOM) and British Columbia, Canada, revealed numerous sequences belonging to gokushoviruses and allowed the assembly of five putative genomes with an organization similar to chlamydiamicroviruses. Fragment recruitment to these genomes from different metagenomic data sets is consistent with gokushovirus genotypes being restricted to specific oceanic regions. Conservation among the assembled genomes allowed the design of degenerate primers that target an 800 bp fragment from the gene encoding the major capsid protein. Sequences could be amplified from coastal temperate and subtropical waters, but not from samples collected from the Arctic Ocean, or freshwater lakes. Phylogenetic analysis revealed that most sequences were distantly related to those from cultured representatives. Moreover, the sequences fell into at least seven distinct evolutionary groups, most of which were represented by one of the assembled metagenomes. Our results greatly expand the known sequence space for gokushoviruses, and reveal biogeographic separation and new evolutionary lineages of gokushoviruses in the oceans.

## Introduction

Viruses are the most abundant (Suttle, 2005) and diverse (Breitbart et al., 2002; Angly et al., 2006) biological entities in the oceans. By causing lysis of specific subsets of microbial communities, they influence community composition by controlling species evenness and maintaining species richness (Hennes et al., 1995; Thingstad, 2000; Wommack and Colwell, 2000; Middelboe et al., 2001; Weinbauer, 2004; Winter et al., 2010); thereby, influencing nutrient and energy cycling (Fuhrman, 1999; Wilhelm and Suttle, 1999; Suttle, 2007). Moreover, viruses harbor an enormous pool of genetic diversity that can be exchanged among other viruses (Pedulla et al., 2003; Short and Suttle, 2005) and bacteria (Fuhrman and Schwalbach, 2003; Kenzaka et al., 2010). Despite the abundance of bacteriophages in marine systems (often >10^7^ ml^−1^) and their important role in marine systems, relatively little is known about the distribution and composition of most groups of marine viruses.

Metagenomic approaches have provided an in-depth look at the molecular diversity of ssDNA viruses in a range of environments including marine systems (Breitbart et al., 2002; Angly et al., 2006; Bench et al., 2007), the human gut (Zhang et al., 2006; Breitbart et al., 2008; Minot et al., 2011), modern stromatolites (Desnues et al., 2008), and freshwaters (Kim et al., 2008; López-Bueno et al., 2009; Roux et al., 2012a). Recently, 608 ssDNA viral genomes were assembled from marine metagenomic data revealing far greater evolutionary diversity in ssDNA viruses than previously known (Labonté and Suttle, 2013).

Gokushoviruses are ssDNA bacteriophages belonging to the family *Microviridae* and are represented among sequences found in metagenomic data. For example, gokushovirus genomes were assembled from a wide range of environments by mining of metagenomic data, with 42 assembled from a variety of ecosystems (Roux et al., 2012b), and two others from data collected from the North Atlantic Ocean (Tucker et al., 2011), indicating the widespread occurrence of gokushoviruses. These viruses have a ~30 nm icosahedral capsid encompassing a positive ssDNA molecule of 4.4 to 4.8 kb that encodes five major proteins. Based on the phylogeny of the major capsid protein (VP1) of isolates, the *Microviridae* are divided into two subfamiles (Brentlinger et al., 2002). Members of the *Microvirinae* (e.g., phiX174 and G4) infect enterobacteria including *Escherichia coli* (Godson et al., 1978), while members of the *Gokushovirinae* infect parasitic bacteria. The latter includes Chp1 (Storey et al., 1989), Chp2 (Liu et al., 2000; Everson et al., 2002) and Chp3 (Garner et al., 2004) that infect *Chlamydia* spp., while phiMH2K (Brentlinger et al., 2002) and SpV4 (Chipman et al., 1998) infect *Bdellovibrio* sp. and *Spiroplasma* sp., respectively.

There are no reported gokushovirus isolates, and their hosts remain unknown. Based on bacterial genomic sequences bacteria in the *Bacteroidetes* appear to be hosts for a proposed subfamily, *Alpavirinae* (Krupovic and Forterre, 2011), of previously unknown microviruses. As well, eight ssDNA phages have been isolated that are morphologically similar but genetically different to microviruses (Holmfeldt et al., 2013, 2012).

Our study examined the genetic diversity and relatedness of *Gokushovirinae*-like viruses from temperate and subtropical coastal environments. From three ssDNA-enriched metagenomic datasets we assembled and phylogenetically compared five new gokushovirus genomes. Recruitment of metagenomic reads to these genomes showed spatial differences in the most abundant gokushovirus genotypes. The genetic richness of gokushoviruses was also assessed through amplification of a ~800 bp fragment of the conserved gene encoding the major capsid protein, VP1. These results reveal biogeographic separation and new evolutionary lineages of marine gokushoviruses, and likely reflect the underlying distributions of their hosts.

## Materials and methods

### Collection and preparation of samples

Samples (~20 to ~200 L) were collected using GO-FLO or Niskin bottles either mounted on a CTD rosette or directly on a hydrographic wire [Saanich Inlet (SI)], or by bucket from the surface (lake samples). For each sample, the viruses were concentrated ~10–100-fold (~200 mL final volume) using ultrafiltration (Suttle et al., 1991). Briefly, particulate matter was removed by pressure filtering (<17 kPa) the samples through 142-mm diameter glass-fiber filters (MFS GC50, nominal pore size 1.2 μm) and polyvinylidene difluoride filters (Millipore GVWP, pore size 0.22 μm) connected in series. The viral size fraction in the filtrate was then concentrated by ultrafiltration though a 30 kDa molecular weight cut-off cartridge (Amicon S1Y30, Millipore), and stored at 4°C in the dark until processed.

In order to integrate variation within a region, numerous virus concentrates (VCs) collected from different locations and at different times within a geographic region were combined into a single mix. Except for the SI and freshwater samples, these mixes corresponded to those used by Angly et al. (2006) in which the first ssDNA viral sequences were reported from marine metagenomic data. VCs from the Strait of Georgia (SOG) and surrounding inlets and bays were pooled into the following four mixes by combining 2 mL of each VC: BC1-1999 (23 samples), BC3-2000 (26 samples), BC4-2004 (16 samples), and BC2-Low salinity (19 samples). Similarly, samples from the Gulf of Mexico (GOM) were combined into four mixes from the eastern GOM (8 samples), northern GOM (6 samples), western GOM (6 samples), and the Texas Coast (13 samples), while samples from the Arctic Ocean were made into mixes from the Beaufort Sea (20 samples), Chukchi Sea (14 samples) and High Arctic (22 samples). To look at the diversity of freshwater gokushoviruses, two mixes were made from Chilliwack (6 samples) and Cultis (8 samples) Lakes. An extensive description of all the samples that were combined in each mix is presented in the Supplementary Material of Labonté and Suttle (2013). SI is unusual as it undergoes seasonal anoxia (Zaikova et al., 2010). For the metagenomic study, we combined surface samples from April 2007, and January, March, May, July, August, and November 2008. PCR amplifications were performed on the following nine samples from SI: 10, 120, and 150 m samples from April 2007, and surface samples from January, March, May, July, August, and November 2008.

### ssDNA preparation

As described in Labonté and Suttle (2013), ssDNA was prepared from 10 mL of 0.22-μm filtered (PDVF; Millipore) pooled mixes from British Columbia (SOG), the GOM, the Lakes (LA), and the Arctic (ARC), or from 10 mL of each individual VCs from SI. Briefly, ssDNA was extracted using a silica column and amplified using multiple-displacement whole-genome amplification (WGA) to convert ssDNA into dsDNA. Pure amplified dsDNA was resuspended in ultrapure H_2_O for pyrosequencing or Tris-HCl for PCR amplification.

### Genome analysis, binning, and assembly

Metagenomic libraries were constructed from WGA ssDNA from SI, SOG, and GOM ssDNA. The purified WGA DNA was resuspended in 100 μL of RNAse- and DNAse-free water (Invitrogen) and concentrated using a Millipore YM-30 Microcon centrifugal filter to a final volume of ~50 μL; 3–5 ug of DNA from each sample was sent for pyrosequencing (Roche 454 FLX instrumentation with Titanium chemistry) at Genome Québec, McGill University (SOG) and the Broad Institute at the Massachusetts Institute of Technology (GOM and SI).

The sequences were quality and linker trimmed, and assembled into contiguous sequences (contigs) using the Newbler Assembler (Roche). The individual reads and assembled sequences were compared to a database of all available genomes in GenBank (as of February 2010) from viruses belonging to the *Microviridae* using the tBLASTx algorithm with an *e*-value cut-off of 10^−5^. Reads with significant similarity to gokushoviruses were aligned onto the assembled contigs using the add454Reads.perl script and were reassembled into new contigs using the phredPhrap.perl script of the Consed package (Gordon, 2003). Additional contig analyses (BLAST, circularization of the genomes, annotations, alignments, and phylogeny) were performed within the Geneious Pro package v5.6 (Biomatters).

### Primer design and PCR amplification

Two forward (MicroVP1-F1, 5′-CGN GCN TAY AAY TTR ATH-3′; MicroVP1-F2, 5′-AGN GCN TAY AAY TTR CTN-3′) and two reverse (MicroVP1-R1, 5′-TTY GGN TAY CAR GAR AGN-3′; MicroVP1-R2, 5′-NCT YTC YTG RTA NCC RAA-3′) primers with respective degeneracies of 256, 215, 256, and 256 were designed from alignments of the inferred amino acid sequences of the major capsid protein (VP1) of the chlamydiaphages Chp1 (accession number NC_001741.1), Chp2 (NC_002194.1), Chp3 (NC_008355.1), Chp4 (NC_007461.1), phiCPAR39 (NC_002180.1), and phiCPG1 (NC_001998.1), Spiroplasmaphage Sp4 (NC_003438.1), bdellovibriophage phiMH2K (NC_002643.1), and the Sargasso Sea Chp1-like assembled genome (Angly et al., 2006; Tucker et al., 2011). The primers amplify a ~800 bp VP1 gene fragment from the subfamily *Gokushovirinae* in the *Microviridae*.

Prior to use in PCR reactions, the purified WGA DNA was resuspended in 100 μL of TE, and 10 μL was used as a template in each PCR reaction mixture consisting of Taq DNA polymerase assay buffer [20 mM Tris·HCl (ph 8.4), 50 mM KCl], 1.5 mM MgCl_2_, 125 μM of each deoxyribonucleoside triphosphate, 1 μM of each MicroVP1-F1, MicroVP1-F2 and MicroVP1-R1 and MicroVP1-R2 primer and 2.5 U of PLATINUM Taq DNA polymerase (Invitrogen). Negative controls contained all reagents except DNA template. The samples were denatured at 94°C for 3 min, followed by 35 cycles of denaturation at 94°C for 30 s, annealing at 50°C for 30 s, and elongation at 72°C for 50 s, with a final elongation step of 72°C for 5 min.

### Clone library construction and RFLP analysis

PCR amplicons were purified with a MinElute PCR purification kit (Qiagen), ligated into pCR2.1-Topo (Invitrogen), and used to transform chemically competent *E. coli* Top10 cells. For each sample, 30 clones were checked by colony PCR to verify that they contained an insert of the correct size. Restriction fragment length polymorphism (RFLP) analysis was then performed on 20 positive clones. For each RFLP reaction, 15 μL of colony PCR product was digested with *AluI* (New England BioLabs) in a reaction containing 1 U/μg of DNA and 1× NEBuffer 4 (20 nM Tris-acetate, 50 mM potassium acetate, 10 mM magnesium acetate, 1 mM DTT, pH 7.9) by incubating at 37°C for 16 h, followed by heat inactivation at 65°C for 20 min. RFLP products were separated on a 2% agarose gel in 0.5× TBE (9 mM Tris base, 9 mM boric acid, 2 mM EDTA, pH 8.0) running at 110 V for ~2 h. Sequencing of representative clones confirmed that each unique restriction pattern could be considered as an operational taxonomic unit (OTU). Forward and reverse sequences (~800 bp) were obtained for each RFLP pattern using Big-Dye Terminator Cycle Sequencing (Applied Biosystems) and ABI 373 Stretch or ABI Prism 377 sequencers (Nucleic Acid Protein Service Unit, UBC).

### Phylogenetic analysis

For the whole genome phylogeny, non-coding sequences were removed and the five major open reading frames were ordered. The sequences were aligned using MAFFT (Katoh et al., 2002) and maximum likelihood analysis with 100 bootstrap replicates were performed using PhyML (Guindon et al., 2010).

VP1 from the previously sequenced isolates, environmental sequences, and the degenerate PCR products from this study were trimmed to the PCR-product length (~800 bp) and aligned using MAFFT (Katoh et al., 2002). The alignment was cured with GBlocks to remove unconserved regions that aligned with multiple gaps using the less stringent setting (allowing for smaller final blocks, gap positions within the final blocks and less strict flanking positions) (Talavera and Castresana, 2007). Bayesian phylogenetic analyses were performed on the cured alignment with MrBayes (Huelsenbeck and Ronquist, 2001). MrBayes uses a Markov chain Monte Carlo (mcmc) approach to approximate prior and posterior probabilities. Under the HKY85 substitution model with an invgamma distribution, two independent analyses of 4 (1 cold and 3 heated) mcmc chains with 20,000,000 cycles were run, sampled every 1000th cycle. The consensus tree was generated in Geneious with a burnin of 25%. Trees were viewed in Fig Tree (http://tree.bio.ed.ac.uk/software/figtree/).

### Fragment recruitment

Recruitment of the reads from metagenomic data sets onto the assembled genomes was performed using tBLASTx with an *e*-value of 10^−10^ and allowing only one hit per read. The metagenomic reads from the marine viromes (Angly et al., 2006) and microbialites (Desnues et al., 2008) were obtained from the CAMERA database, while the metagenomic reads from Lake Pavin and Lake Bourget (Roux et al., 2012a) were obtained from the SEED database. The environmental genomes used were Lake_Bourget_052, Lake_Bourget_523, Lake_Pavin_279 and 68_Microbialite_063 from Roux et al. (2012b), and SARssphi2 from Tucker et al. (2011).

### Nucleotide sequence accession numbers

The five complete gokushovirus genomes as well as the 43 environmental PCR product sequences were submitted to Genbank and are available under the accession numbers KC131021-KC131025 and KC130978-KC131020, respectively.

## Results and discussion

### Assembly of complete gokushovirus genomes

**Figure 1:**
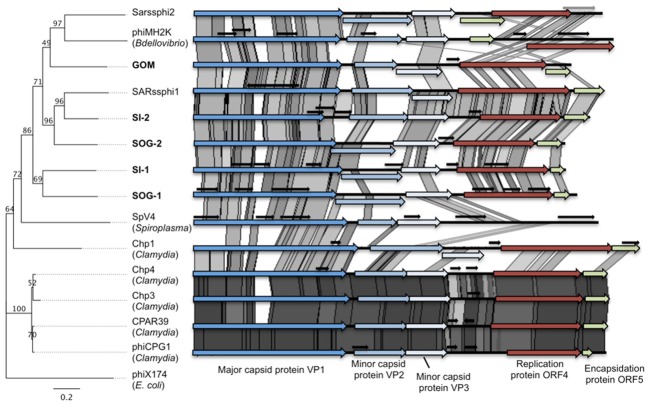
**Gokushoviruses share a similar genome organization.** Whole genome phylogeny (Maximum likelihood, 100 bootstrap replicates, HKY85 model) on the ORFs of gokushoviruses rooted with the microvirus phiX174 (left) and pairwise comparisons of the five environmental gokushovirus genomes assembled from this study (bold) with the isolates and other environmental genomes. Conserved genes are represented by colored arrows, while small overlapping genes of unknown function are represented by short black arrows. The genome similarities were visualized in ACT (Carver et al., 2005) (*e*-value <10^−5^) and the gray shading indicates the level of similarity; darker shading represents higher similarity between pairs of ORFs.

Sequence analysis of ssDNA metagenomic libraries from the SOG, SI, and the GOM recovered 1733, 374, and 194 sequences, respectively, that were significantly similar to sequences from viruses belonging to the *Microviridae*, with >90% of them being most similar to sequences belonging to the chlamydiamicroviruses and other gokushoviruses. From these data, five complete circular genomes were assembled with at least 3-fold coverage (two from SOG, two from SI and one from GOM). The genome sizes varied from 4062 to 5386 bp, and were uniformly shorter than those from previously sequenced isolates (Figure 1). Assembly of these genomes represented the accumulation of 95 reads for SOG-1, 58 for SOG-2, 53 for SI-1, 48 for SI-2, and 38 for GOM.

Even though there was only ~30–50% similarity at the nucleotide level among the assembled genomes (Table 1), the chlamydiaphages and bdellovibriophage phiMH2K, the gene organization was remarkably similar among them, and included the five proteins required for replication of gokushoviruses (Figure 1), implying a common evolutionary origin. These comprise VP1, the major capsid protein, VP2 that is hypothesized to be involved in host recognition (Chipman et al., 1998) and virus attachment (Everson et al., 2003), VP3 that is a scaffolding protein found in the procapsid only and not in mature virions (Clarke et al., 2004), ORF4 that is a replication initiator involved in ssDNA synthesis (Liu et al., 2000; Garner et al., 2004; Salim et al., 2008), and ORF5 that is involved in DNA packaging (Liu et al., 2000; Garner et al., 2004; Salim et al., 2008). The presence of all five essential genes in the assembled genomes strongly suggests they represent complete sequences from extant viruses in the environment.

**Table 1:**
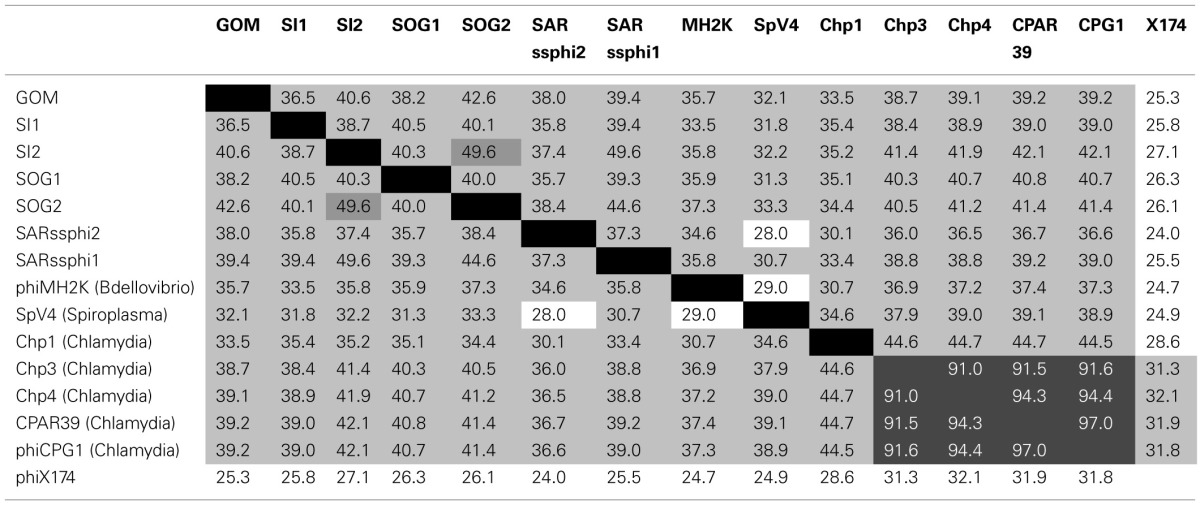
**Similarity matrix of the coding regions of the five environmental gokushovirus genomes assembled from this study (bold) with the isolates and other environmental genomes**.

*(i.e.* 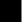 >*90%*, 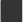 *45–89%*, 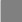 *30–45%*, and 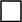 >*30%).*

Whole genome phylogeny revealed that the environmental genomes cluster more closely with the bdellovibriophage phiMH2K, rather than the chlamydiaphages (Figure 1), suggesting that the host for these gokushoviruses is more closely related to *Bdellovibrio* spp., which are found in marine waters, than *Chlamydia* spp. Whole genome pairwise comparisons showed that VP2 and ORF4 are the least conserved genes, with very few regions of conservation. Moreover, there is 91–97% similarity among chlamydiamicroviruses, while only 28–49% similarity among the environmental phages. A recombination event in which ORF4 and ORF5 are inverted in phiMH2K, which infects the bacterial parasite *Bdellovibrio bacteriovorus*, and in the environmental genome SAR phi2. These genomes also cluster together suggesting a common evolutionary history (Figure 1).

All of the environmental genomes were shorter than those from isolates. Some, such as SI-1 and SOG-1, had multiple overlapping genes of unknown function. It is postulated (Rokyta and Burch, 2006) that ssDNA microviruses, such as the coliphages phiX174 and G4, evolve differently than dsDNA viruses because of strictly lytic life cycles, small genomes, and low rates of horizontal gene transfer (Breitbart and Rohwer, 2005; Comeau and Buenaventura, 2005; Hambly and Suttle, 2005). Novel genes were predicted to originate by overprinting rather than by horizontal gene transfer (Pavesi, 2006).

### Genetic relatedness among genes encoding the major capsid protein

To look more deeply at the genetic richness of gokushoviruses, degenerate primers were designed to amplify a ~800 bp fragment of the gene encoding the major capsid protein (VP1) that has interspaced conserved and variable regions. For the assembled genomes, the phylogeny of VP1 is congruent with the whole genome; thus, the phylogeny of VP1 can be used to infer viral phylogeny. PCR amplification was performed on samples from the SOG (4 mixes), GOM (4 mixes), SI (9 samples), and Arctic (ARC; 3 mixes) (Table S1). No products were amplified from the ARC, LA, SOG-Low Salinity, or Eastern GOM mixes. This means that gokushoviruses were absent or at low concentrations in these samples, or that they are too divergent to be amplified by the primers.

**Figure 2:**
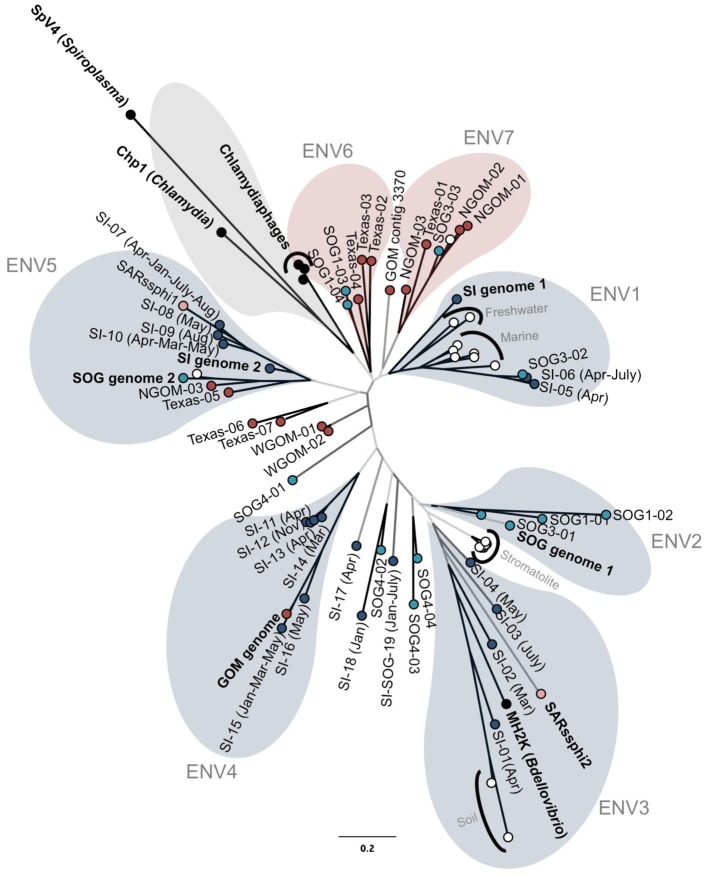
**Genetic relatedness of the major capsid protein gene VP1.** Unrooted Bayesian phylogenetic analysis (20 million MCMC generations with 25% burnin; HKY85 model) of PCR products from GOM (red), Strait of Georgia (light blue), Saanich Inlet (dark blue), cultured isolates (black), and other environmental sequences (white). Bootstrap support of at least 90%, 75% and less than 75% is represented by black, dark gray, and light gray branches, respectively. Colored bubbles represent the different supported sub-groups of gokushoviruses with more than two sequences containing a complete sequenced environmental genome (blue), only isolates (gray), or only PCR products (red). The scale bar represents 0.2 nucleotide changes per site.

Twenty VP1 clones from each of the 20 samples were digested using *AluI* to reveal 77 different RFLP patterns. Representative clones from each restriction pattern were sequenced (data not shown). Of these, 43 sequences were at least 98% different at the nucleotide level, and thus identified as unique gokushovirus VP1 sequences. Some sequences occurred in more than one sample from the same geographic region; for example, the sequence SI-07 was sequenced from multiple dates in SI (i.e., January, April, May and Aug), but no sequences occurred in more than one location (Figure 2). Some sequences were found in both SOG and SI, but no sequences were found in GOM and SOG, or GOM and SI. The 85 % of the VP1 sequences that contained the primer sequences and translated into a putative protein were kept for further analysis. The nucleotide alignment revealed multiple regions of conservation, as well as regions that were confined to specific groups, agreeing with observations made during primer design.

The amplified VP1 PCR products were compared to VP1 sequences containing the primer sequences from the assembled genomes, the chlamydiamicrovirus isolates, as well as Genbank environmental sequences from modern stromatolites (Desnues et al., 2008), freshwater (Lake Needwood, MD; Kuzmickas et al., unpublished), rice paddy soil (Kim et al., 2008) and marine genomes (Venter et al., 2004). Few sequences were similar to those from isolates. Phylogenetic analysis using Bayesian (Figure 2) and maximum-likelihood algorithms produced similar trees with gokushoviruses sub-divided into at least seven well-supported new groups containing more than two sequences (Figure 2). Five of the new clades are represented by an assembled genome or sequenced isolate (Figure 2). Several sequences, such as SOG4-04 and SI-18, were too divergent to be assigned to a cluster; however, as only 20 clones were analyzed for each sample, rarer phylogenetic clusters were poorly sampled.

Sequences from a given location were usually more closely related to ones from the same location; most GOM sequences clustered within ENV6 and ENV7, while ENV2 is represented exclusively by SOG sequences. Sequences found in more than one sample also usually clustered together. For example, the sequences SI-10 and SI-07, which were found in SI on multiple dates, clustered within ENV5, along with sequences from GOM and SI-2. Collectively, these data imply that viruses in the ENV5 group are widespread in nature. Other data from modern stomatolites and marine genomes clustered together as specific phylogenetic groups.

### Host specificity and geographic distribution of environmental gokushovirus genomes

**Figure 3:**
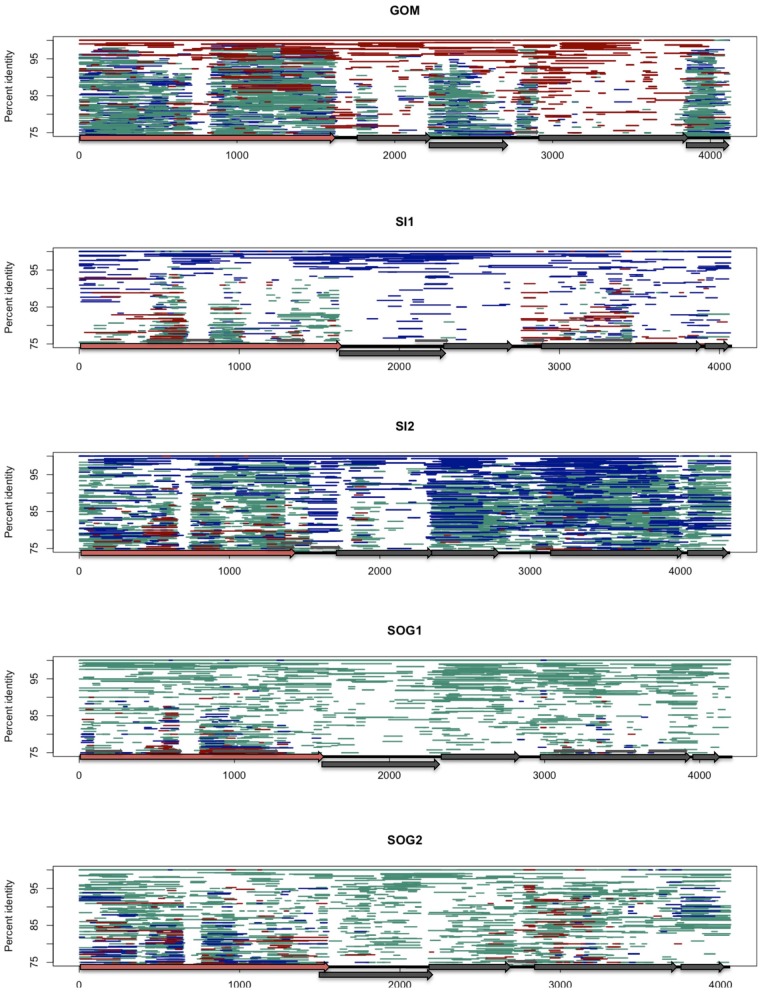
**Fragment recruitment of the viral ssDNA reads on the genomes from this study to show regions of conservation among ssDNA gokushoviruses.** Each assembled genome (GOM, SI1, SI2, SOG1, SOG2) is represented by a different panel. Each horizontal line represents a metagenomic read from ssDNA data sets from the Gulf of Mexico (dark red), Saanich Inlet (Dark blue), and Strait of Georgia (aqua) on each of the assembled genomes. Reads were recruited using tBLASTx with an *e*-value of 10^−10^. The position of each line represents the percent similarity of the read to the genome.

**Figure 4:**
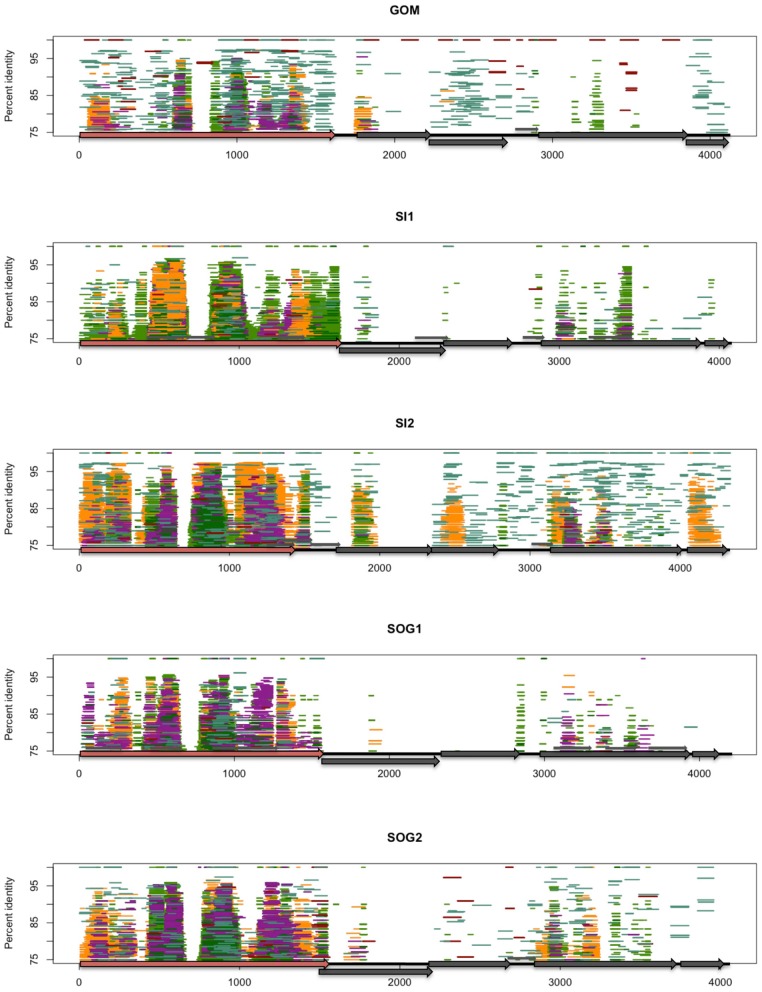
**Fragment recruitment of reads from environmental viral metagenomes to show the regions of conservation within different environments.** Each assembled genome (GOM, SI1, SI2, SOG1, SOG2) is represented by a different panel. Each horizontal line represents a read recruited from one of the following publicly available metagenomic data sets: Gulf of Mexico (dark red), Strait of Georgia (aqua), Sargasso Sea (orange), Lake Bourget (light green), Lake Pavin (dark green), and microbialites (purple). Reads were recruited against each of the assembled genomes using tBLASTx with an *e*-value of 10^−10^. The position of each line represents the percent similarity of the read to the genome. VP1 is represented by a red arrow.

Isolates in the *Gokushovirinae* infect parasitic bacteria, such as *Chlamydia* spp., *Bdellovibrio* spp., and *Spiroplasma* spp., with host specificity likely being dictated by variable genomic regions. To investigate conserved and variable motifs, metagenomic reads from our ssDNA data, as well as other viral metagenomic data sets from marine (Angly et al., 2006), freshwater (Roux et al., 2012a), and microbialite (Desnues et al., 2008) environments were recruited against environmental gokushovirus genomes (Figure 3). Recruitment was more even when the reads were recruited against genomes assembled from metagenomic data collected from the same region (Figures 3, 4; Figures S1, S2). For the SOG-1, SOG-2, and SI-2 genomes, few reads were recruited from data sets other than those from which the genomes were assembled, suggesting that these genomes are not widespread (Figure 3). In contrast, reads from all of the metagenomic data sets aligned on the GOM genome (Figure 3), indicating a wider geographic distribution of these viruses. The high level of recruitment from other data sets on the GOM genome is also congruent with the phylogenetic clustering of the VP1 gene with other VP1 sequences that were present in multiple samples (Figure 2).

The distribution of reads from other environmental samples that recruited to the assembled genomes was very uneven, showing regions of higher conservation within VP1 and VP3, while few reads were recruited to the VP2 region, indicating high variability in this gene (Figure 4). Since the VP2 sequences of the assembled genomes differ from those of isolates, and VP2 encodes for the minor capsid protein involved in host recognition (Chipman et al., 1998), the environmental sequences are likely not from viruses infecting the genera *Chlamydia*, *Bdellovibrio*, or *Spiroplasma*. Recruitment to ORF4 was limited to the source environment for the assembled genomes, and metagenomic data from British Columbia were not recruited to ORF4 of the GOM genome. Thus, both the pairwise comparison (Figure 1) and the recruitment of metagenomic reads (Figure 3) showed that VP2 and ORF4 are less conserved. Similar patterns were observed with genomes assembled from other data sets (Figures S1, S2), showing that specific gokushovirus genotypes are restricted in distribution.

No sequences similar to gokushoviruses were amplified from the Arctic Ocean or two lakes in British Columbia. However, gokushovirus sequences have been found in an Antarctic lake (López-Bueno et al., 2009) and other freshwater environments (Roux et al., 2012a), suggesting that freshwater gokushoviruses differ enough in sequence that they cannot be amplified using our primers.

### Possible role of gokushoviruses in aquatic environments

The distribution of gokushovirus OTUs with respect to specific marine environments differs from observations that some viral genotypes are widely distributed Chen and Suttle, 1996; Fuller et al., 1998; Hambly et al., 2001; Short and Suttle, 2002, 2005; Breitbart et al., 2004; Labonté et al., 2009. However, Tucker et al. (2011) observed differences in the depth distribution of gokushovirus sequences in the North Atlantic Ocean that likely reflected the distribution of hosts. Most VP1 sequences that were found in more than one sample were also relatively closely related, perhaps reflecting viruses that have a broader host range, or viruses that infect widely distributed hosts. In contrast, sequences specific to a single location are probably from viruses that infect bacteria that are environment specific. Although some bacterial species are very widely distributed (Rusch et al., 2007; Biers et al., 2009), others are restricted to specific habitats (Biers et al., 2009). Hence, it is not surprising that some gokushoviruses have a very restricted distribution.

Based on previous work (Labonté and Suttle, 2013), gokushovirus sequences were not the most abundant viruses in our samples, and comprised only 1.6, 0.4, and 0.2% of the metagenomic reads from the SOG, SI, and GOM, respectively. In contrast, in metagenomic data from the Sargasso Sea, gokushovirus sequences comprised nearly 6% of the reads (Angly et al., 2006), while in Lake Bourget they were more than 90% of the sequences (Roux et al., 2012a). These differences may be because the small genomes of gokushoviruses permit rapid replication and high burst sizes, and allow them to dominate following a lytic event, this is consistent with the hypothesis that the most abundant marine viruses are virulent opportunists that replicate rapidly, have high burst sizes and small genomes in order to exploit rapidly growing populations of rare marine bacteria such as *Roseobacter* spp. or *Vibrio* spp. (Suttle, 2007). For example, ~500 genomes are produced each time the chlamydiaphage Chp2 infects its parasitic host (Salim et al., 2008). High burst sizes coupled with genomes usually <5 kb support the idea that gokushoviruses are highly virulent and are selected for rapid population growth, which are characteristics of r-strategists. In contrast, many large DNA viruses have a low burst size, large genome and decay slowly, which are characteristics of K-strategists.

Discovering the hosts of marine gokushoviruses is a high priority in order to understand the roles that these viruses play in ecosystems. Given the challenges in culturing marine microbes, culture-independent techniques will likely be needed to determine the hosts for most of these viruses. One approach that we have tried with some success is to use fluorescence *in situ* hybridization (FISH) using labeled VP1 sequences to probe natural microbial communities. Another approach that has been used to visualize phage-infected gammaproteobacterial cells is phageFISH (Allers et al., 2013), which could be adapted to search for cells infected by gokushoviruses. Finally, single-cell genomics (SCG) allows everything in a cell, including plasmids and viruses to be sequenced (Stepanauskas, 2012). If applied to samples with abundant gokushoviruseses, it should be possible to sequence infected cells.

This manuscript presents a new set of degenerate primers that have been used to reveal at least five new evolutionary groups of gokushoviruses, and clearly show they share a common evolutionary history with viruses that infect the obligate intracellular parasitic bacteria *Chlamydia* and *Bdellovibrio*. Phylogenetic analysis of the major capsid protein, combined with fragment recruitment of environmental metagenomic sequences shows that the distribution of some evolutionary groups of gokushoviruses is very environment dependent, whereas others are more cosmopolitan. The high-burst size, rapid replication rates and likely lytic nature of these viruses suggests that they may play an important role as mortality agents in marine systems.

## Conflict of interest statement

The authors declare that the research was conducted in the absence of any commercial or financial relationships that could be construed as a potential conflict of interest.

## Supplementary material

Figure S1 **Fragment recruitment of viral ssDNA reads onto assembled environmental gokushovirus genomes.** Each assembled genome (Lake_Bourget_052, Lake_Bourget_523, Lake_Pavin_279, 68_Microbialite_063, and SARssphi2) is represented by a different panel. Each horizontal line represents a read recruited from one of the metagenomic data sets from this study: Gulf of Mexico (dark red), Saanich Inlet (Dark blue), and Strait of Georgia (aqua). Reads were recruited against each of the assembled genomes using tBLASTx with an *e*-value of 10^−10^. The position of each line represents the percent similarity of the read to the genome. VP1 is represented by a red arrow.

Figure S2 **Fragment recruitment of reads from environmental viral metagenomes to show the regions of conservation within different environments.** Each assembled genome (Lake_Bourget_052, Lake_Bourget_523, Lake_Pavin_279, 68_Microbialite_063, and SARssphi2) is represented by a different panel. Each horizontal line represents a read recruited from one of the following publicly available metagenomic data sets: Gulf of Mexico (dark red), Strait of Georgia (aqua), Sargasso Sea (orange), Lake Bourget (light green), Lake Pavin (dark green), and microbialites (purple) metagenomic data sets on each of the assembled genomes that recruited at using tBLASTx with an *e*-value of 10^−10^. The height of line represent the percent similarity of the read to the genome. VP1 is represented by a red arrow.
