# Corrigendum: Metagenomic and whole-genome analysis reveals new lineages of gokushoviruses and biogeographic separation in the sea

**DOI:** 10.3389/fmicb.2015.00114

**Published:** 2015-02-11

**Authors:** Jessica M. Labonté, Curtis A. Suttle

**Affiliations:** ^1^Department of Microbiology and Immunology, University of British ColumbiaVancouver, BC, Canada; ^2^Department of Earth, Ocean and Atmospheric Sciences, University of British ColumbiaVancouver, BC, Canada; ^3^Department of Botany, University of British ColumbiaVancouver, BC, Canada; ^4^Integrated Microbial Diversity, Canadian Institute for Advanced Research, University of British ColumbiaVancouver, BC, Canada

**Keywords:** biogeography, ssDNA viruses, *Microviridae*, *Gokushovirinae*, virus diversity, ocean viruses

The primer MicroVP1-R1 should read as “5′-NCG YTC YTG RTA NCC RAA-3′.”

## Conflict of interest statement

The authors declare that the research was conducted in the absence of any commercial or financial relationships that could be construed as a potential conflict of interest.

