# Supplementary figures and images for: Metagenomic and whole-genome analysis reveals new lineages of gokushoviruses and biogeographic separation in the sea
Source: Front Microbiol. 2013 Dec 24;4:404. doi: 10.3389/fmicb.2013.00404 (PMC3871881; doi:10.3389/fmicb.2013.00404)

**Bourget\_052**

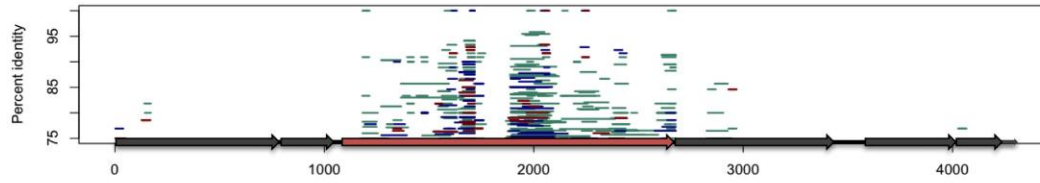

**Bourget\_523**

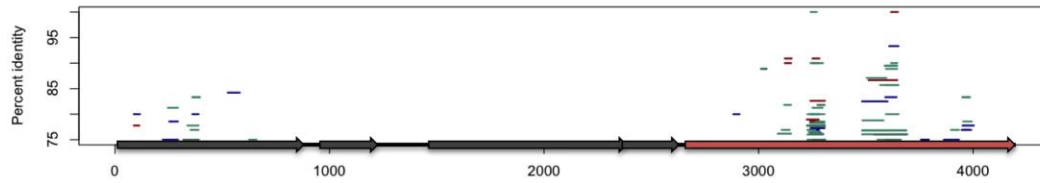

**Pavin\_279**

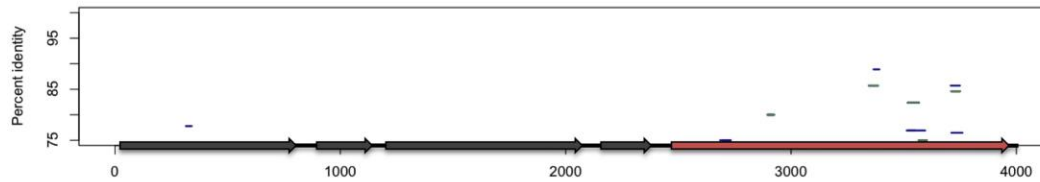

**68\_Microbialite\_063**

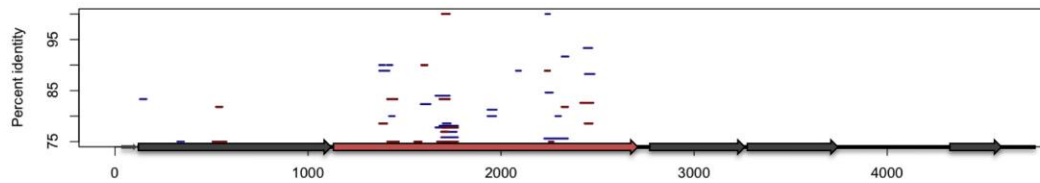

**SARssphi2**

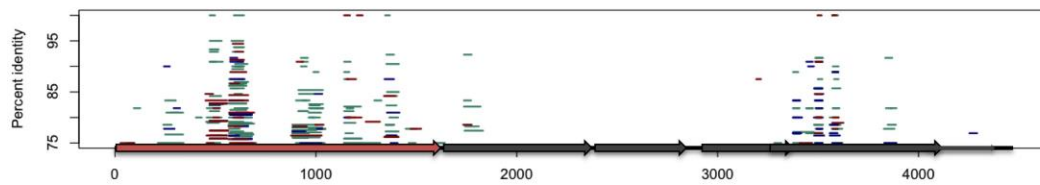

Supplement: Figure S1 — Fragment recruitment of viral ssDNA reads onto assembled environmental gokushovirus genomes. Each assembled genome (Lake_Bourget_052, Lake_Bourget_523, Lake_Pavin_279, 68_Microbialite_063, and SARssphi2) is represented by a different panel. Each horizontal line represents a read recruited from one of the metagenomic data sets from this study: Gulf of Mexico (dark red), Saanich Inlet (Dark blue), and Strait of Georgia (aqua). Reads were recruited against each of the assembled genomes using tBLASTx with an e-value of 10−10. The position of each line represents the percent similarity of the read to the genome. VP1 is represented by a red arrow. [file Presentation1.PDF]

**Bourget\_052**

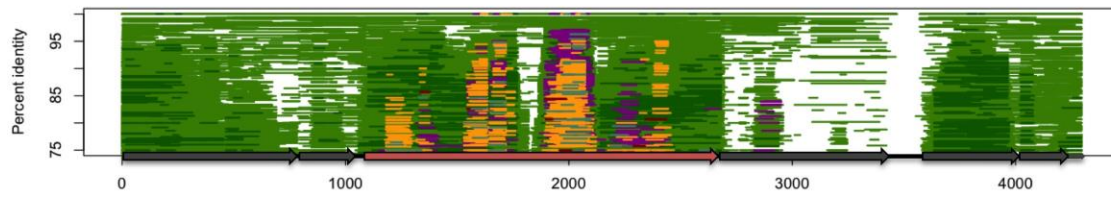

**Bourget\_523**

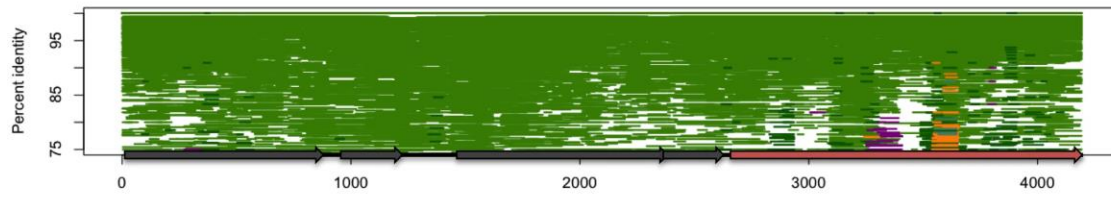

**Pavin\_279**

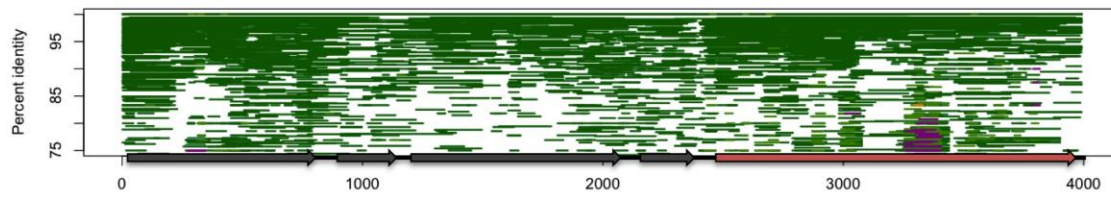

**68\_Microbialite\_063**

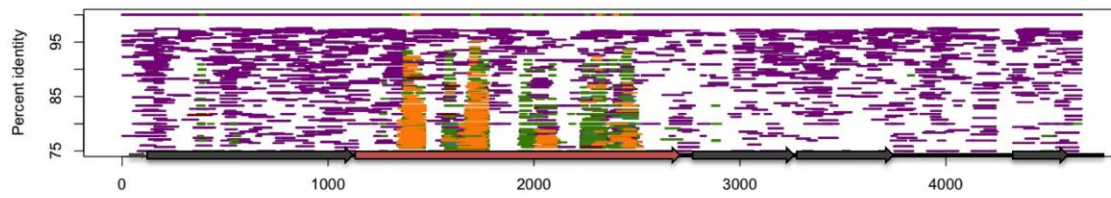

**SARssphi2**

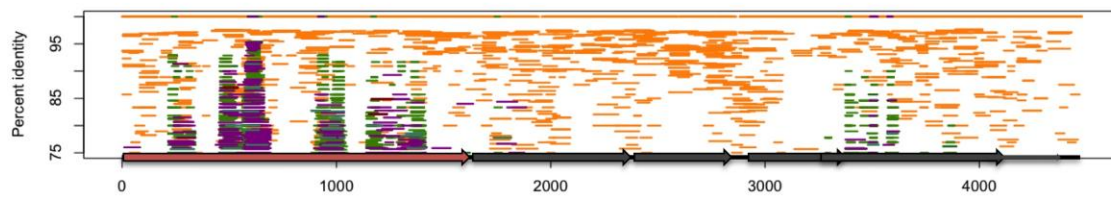

Supplement: Figure S2 — Fragment recruitment of reads from environmental viral metagenomes to show the regions of conservation within different environments. Each assembled genome (Lake_Bourget_052, Lake_Bourget_523, Lake_Pavin_279, 68_Microbialite_063, and SARssphi2) is represented by a different panel. Each horizontal line represents a read recruited from one of the following publicly available metagenomic data sets: Gulf of Mexico (dark red), Strait of Georgia (aqua), Sargasso Sea (orange), Lake Bourget (light green), Lake Pavin (dark green), and microbialites (purple) metagenomic data sets on each of the assembled genomes that recruited at using tBLASTx with an e-value of 10−10. The height of line represent the percent similarity of the read to the genome. VP1 is represented by a red arrow. [file Presentation2.PDF]
